# Canine Traditional Laboratory Tests and Cardiac Biomarkers

**DOI:** 10.3389/fvets.2020.00320

**Published:** 2020-06-26

**Authors:** Alessandra Gavazza, Alessandro Fruganti, Vanessa Turinelli, Andrea Marchegiani, Andrea Spaterna, Beniamino Tesei, Giacomo Rossi, Matteo Cerquetella

**Affiliations:** ^1^School of Biosciences and Veterinary Medicine, University of Camerino, Camerino, Italy; ^2^IDEXX Laboratories-Italia S.r.l, Milan, Italy

**Keywords:** laboratory test, biomarker, cardiac disease, dog, review

## Abstract

In small animals, cardiac disease evaluation through laboratory tests can be a challenge. This review will present both historical and updated perspectives on the clinical pathology of cardiac diseases in dogs and demonstrate that laboratory tests are useful tools for the management of patients with cardiac diseases.

## Introduction

“Traditional” tests such as biochemical, hematological, and coagulative profile, urine and feces analysis, blood culture, and effusion analysis may be altered by cardiovascular diseases but are not specific. On the other hand, of great interest is the study of new and specific tests or biomarkers that could provide information about myocardial injury, to be used in investigating and monitoring patients with cardiac disease. In the last few years, specific hematologic biomarkers such as troponin and natriuretic peptides have been studied; similarly, innovative studies concerning fecal proteomics and microbiota in dogs with cardiac disease were published as well.

Results of laboratory tests have to be analyzed considering clinical evolution, clinical signs, and other specific diagnostic evaluations, in order to use a multimodal approach in the evaluation of the pathology and also of the possible complications of any patient.

## Traditional Laboratory Test

“Traditional” parameters of heart damage are those routinely used for general purposes and are therefore not specific, but they can be useful to confirm a cardiopathy and for monitoring and prognostic purposes.

### Biochemical Profile

Creatine kinase (CK), mainly a cytosolic enzyme, is primarily active in skeletal and cardiac muscles and the brain. Three main isoenzymes (CK-BB, CK-MB, CK-MM) can be separated electrophoretically, but only CK-MB is found in the myocardium. Consequently, the increase in total CK is typically indicative of total muscle damage, not only relative to CK-MB, and unfortunately, plasma CK half-life is under 3 h (1, 2).

Serum aspartate aminotransferase (AST) is a cytosolic and mitochondrial isoenzyme not specific to a tissue; the muscle and liver are its main sources. The amount of the heart muscle is less than that of the skeletal muscle or hepatocyte, and its plasmatic half-life is ~12 h in dogs (1, 2). Lactate dehydrogenase (LDH) is a ubiquitous cytosolic enzyme, although muscle, liver, and erythrocyte damages could be responsible for an increase in serum activity. LDH has five isoenzymes (LDH1, LDH2, LDH3, LDH4, and LDH5) separable by electrophoresis, and LDH1 is most represented in the heart and kidney. Unfortunately, electrophoretic separation is not routinely done in daily practice and therefore total LDH is usually dosed. LDH half-life is < 6 h (1, 2). C-reactive protein (CRP) is considered a major acute-phase protein in dogs, and its amount increases in response to inflammation. In humans, CRP is a mediator of inflammation and an indicator of phlogosis. CRP was analyzed for its relation to atherosclerosis, coronary artery disease (CAD), acute coronary syndromes, and heart failure (HF), and it is now considered to play a role in growth and development of HF (3, 4). CRP in dogs may represent a screening test for a lot of conditions, showing high sensitiveness even if not specific in identifying the underlying pathology. It increases in 4 h following the damage in the dog and reaches a maximum peak in 24–48 h (5). CRP is possibly an indicator of pulmonary hypertension (PH) and endothelial arteritis in canine patients presenting filariasis, and CRP amount might get higher in dogs with dilated cardiomyopathy (DCM) even earlier than lung vascular alterations are evidenced by thorax radiography and echocardiography. Dogs not presenting filariasis evidenced significantly increased CRP rates when suffering from DCM or PH (6). With regard to electrolytes and minerals, hyperphosphatemia is associated with reduced glomerular filtration rate (GFR), and in patients suffering from chronic renal failure, the degree of hyperphosphatemia is directly correlated with the degree of azotemia, which may be related to cardiorenal syndrome (CRS). Hypercalcemia is infrequent in nephropathic dogs (7). Likewise, hypokalemia can develop secondary to significant cardiac disease for translocation across the cell membrane brought about by sympathetic stimulation and for renal loss secondary to high aldosterone concentrations. This latter reason is particularly common while treating HF with diuretics (8, 9). An acute decrease in renal function with oliguria may instead cause hyperkalemia and acidosis because of impaired renal excretions of cations (K^+^ H^+^) (10). On the other hand, the most common cause of moderate to marked hyponatremia in dogs is advanced congestive HF with or without concomitant diuretic therapy (9).

#### Cardiorenal Syndrome

“CRS” has been reported in humans as “*disorders of the heart and kidneys whereby acute or chronic dysfunction in one organ may induce acute or chronic dysfunction of the other*” (11–13). The CRS Consensus Group attempted to reach a consensus in defining canine “cardiovascular-renal disorders” (CvRD) as well as in interpreting the pathophysiology, diagnosis, and management of the condition (14). The cardiorenal axis is the bond between canine chronic heart failure (CHF) and renal insufficiency (RI). When the presence of non-protein nitrogenous compounds, mainly urea and creatinine, increases in blood, it is reported as azotemia (15). Azotemia is high in dogs suffering from chronic valvular disease (CVD), and the risk of azotemia increases with HF severity. The correlation found between ACVIM and both blood urea nitrogen (BUN) and creatinine (CREA) is of clinical importance while treating patients with serious heart disease. Whether the decline in renal function is the consequence of cardiac disease evolution must still be demonstrated (16, 17).

The determination of BUN, CREA, and symmetric dimethylarginine (SDMA) together with urinalysis is necessary for the identification of CRS. Urea is a product of protein metabolism and is excreted via the renal system; therefore, in case of shock, dehydration, cardiovascular disease, etc., in which the GFR decreases, it results in a growth of BUN concentration (10).

CREA is filtered by the glomerulus, and there is no tubular reabsorption. Serum creatinine is a better marker than BUN for GFR, as it is not reabsorbed and is minimally secreted in tubules; obviously, a reduced GFR affects CREA similarly to BUN (10). Finally, SDMA is a sensitive and specific marker of renal function. Serum SDMA levels increase as kidney function and GFR decline. SDMA evaluations in patients suffering from chronic kidney disease (CKD) demonstrated that its concentration increases in dogs up to months earlier than CREA; it, in fact, increases when the GFR is reduced up to 40%, whereas CREA level increases later, with GFR reduction higher than 75% (18).

### Hematological Profile

In human, the contemporary presence of HF, RI, and anemia determines a clinical triangle called *cardiorenal anemia syndrome* in which HF and RI can adversely affect each other and their mutual worsening increased with anemia (19). The pathophysiology of anemia related to HF is caused by many factors and includes renal imbalance and reduced erythropoietin production (19), increased production of proinflammatory cytokines like interleukins and tumor necrosis factor (20, 21), increase in plasma volume (22), and lower production of erythropoietin (23).

In dogs, a study that includes 114 cases of mitral valve disease (MVD) revealed that hemoglobin (Hb) amount and packed cell volume (PCV) reduced with growing seriousness of the condition and that anemia was related with a worse result, emerging as a forecast of mortality. Anemia was also related with HF seriousness and blood creatinine increase, leading the authors to hypothesize the existence of a cardiorenal anemia syndrome similar to what happens in humans (24).

Another study observed in dogs with CVD showed that Hb was negatively correlated with CREA and International Renal Interest Society stage, confirming the existence of a link between renal impairment degree and anemia, but no correlation was found between anemia and classes of HF (16, 25).

Red blood cell distribution width (RDW) quantitatively measures anisocytosis, and in humans it is considered a widely available marker, able to predict adverse outcomes in left-sided HF (26, 27). RDW is also related with fatal outcome in patients with PH. In a recent review, it was suggested that augmented RDW value is related with acute coronary syndrome, cerebrovascular ischemia, and peripheral artery disease, as well as atrial fibrillation, hypertension, and HF (28). Furthermore, a high level of anisocytosis is an important prediction of worse results in patients with these conditions (28). In man, it has been shown in 162 subjects, 62% having PH, that RDW turned out to be related with fatal outcome if serious PH was also present and that it worked more properly as a prognostic indicator rather than the N-terminal fragment of pro B-type natriuretic peptide (NT-proBNP) (see later) (29).

In dogs, a study compared RDW in 44 patients suffering from PH due to different causes; more precisely, post-capillary hypertension was present in 16 of them (caused by left-sided cardiac disease) and pre-capillary hypertension in 28 (caused by primary respiratory disease, pulmonary vascular disease, and idiopathic forms). It demonstrated that RDW is highly different among dogs suffering from pre-capillary PH and controls; however, no significant differences were found between patients showing post-capillary PH and controls. There was a similar proportion of survival rate among dogs gathered depending on RDW values, but RDW did not properly predict PH seriousness (30).

Chronic cardiac and pulmonary disease in dogs, resulting in dyspnea from impaired oxygen transfer, may lead to an appropriate secondary erythrocytosis (31, 32). In physiologically appropriate erythrocytosis, the erythropoietin-mediated increased erythrocyte mass expands the hematic oxygen-carrying capacity in attempt to improve inadequate tissue oxygenation. Congenital heart anomalies, e.g., tetralogy of Fallot, cause delivery of poorly oxygenated blood to the systemic circulation with resultant erythrocytosis (32, 33). In addition, in canine patients presented with congestive heart failure, it was shown that poikilocytosis, which is a general term for variation in RBC shape, resulted from oxidative damage; it is a non-specific observation as occurring in a variety of conditions. In particular, in dogs with congestive heart failure (and glomerulonephritis), keratocyte (spiculated RBCs that frequently have only 1-2 spicules and indicate RBC fragmentation) and schistocyte (red blood cell fragments usually resulting from direct physical damage to RBCs) can be found (34, 35).

Congenital dyserythropoiesis, polymyopathy, and cardiac disease (cardiomegaly) were reported in three related English Springer Spaniels. All dogs presented microcytic normochromic non-regenerative anemia associated with marked metarubricytosis. Alterations in RBC morphology such as spherocytes, schistocytes, dacryocytes, codocytes, and vacuolated RBC were also present (36).

Also, changes in white blood cell type and number may be seen in inflammatory cardiovascular disease. Bacterial endocarditis is typically associated with leukocytosis, monocytosis, neutrophilia, and a left shift. Leukocytosis and thrombocytopenia have been reported to be present in nearly 90% of patients in a case series of dogs with inflammatory cardiovascular disease (37).

### Coagulative Profile

People with chronic heart failure show increased thrombin activity, fibrinolytic activity, and platelet activation (38, 39). In a study involving canine patients suffering from CHF, mainly Cavalier King Charles Spaniels, fibrinogen, D-dimer, and thrombin-anti-thrombin complex concentrations, contrary to anti-thrombin and protein C activity, were, respectively, increased and lowered compared with controls. This data suggests the presence of a procoagulant and overt thromboembolism state in CHF cases. The five hemostatic biomarkers abovementioned were not found to be predictors of mortality (40).

In addition, MVD is related with enhanced reactivity and decreased persistence of platelets in patients, and a possible association between MVD and platelet dysfunction has also been investigated in dogs, particularly in Cavalier King Charles Spaniels. One study carried out in two subgroups of Cavalier King Charles Spaniels presenting prolapsing mitral valves evidenced that one group, which comprehends <100.000 platelets/μL, had usual platelet aggregation and enlarged platelets, while the second group, which contains more than 100.000 platelets/μL, showed augmented platelet aggregation and standard platelet size (41).

Another study on Cavalier King Charles Spaniels with various grades of mitral valve regurgitation showed that dogs may have a decreased platelet function but not an increased platelet reactivity. The structure of the mechanism behind the reduction in platelet activity is still not clear, but an activation followed by a deactivation of platelets, due to recurrent high shear stress, may represent the underlining cause (42). A further study found that Cavalier King Charles Spaniels with mitral valve prolapse had enhanced aggregation responses regardless of mitral regurgitation status, but platelets were not circulating in an activated state. In that study, Cavalier King Charles Spaniels with mitral regurgitation (MR) and dogs presenting subaortic stenosis had prolonged platelet function tests [platelet function analyzer (PFA) −100 closure times]. Also, they had lower proportions of large von Willebrand factor (VWF) multimers suggesting a mechanism of impaired primary hemostasis, different from platelet hypofunction (43).

### Urinalysis

This exam is useful to understand the degree of involvement of the heart and kidneys in the presence of a CRS. In cardiorenal axis, the existing link among CHF and RI in dogs could be confirmed by urinalysis. Urine-specific gravity (USG) determined by refractometry allows assessment of renal concentrating ability. Interpretation of CREA in combination with USG can help to determine whether azotemia is renal or non-renal. However, it is frequently difficult to judge whether elevation of urea and/or creatinine in patients with heart disease is due to intrinsic renal disease or pre-renal azotemia. USG in azotemic patients with cardiovascular disease not undergoing any therapy may help to differentiate concurrent primary renal disease from pre-renal azotemia as consequence of reduced blood flow. In such a case of pre-renal azotemia, USG is usually > 1,030, while when azotemia is renal, the USG is usually between 1,007 and 1,013. Unfortunately, USG cannot be used in many patients to distinguish between these two conditions because they are often receiving diuretics, which reduces its value (7, 8, 10).

Proteinuria must be interpreted simultaneously to USG. Measuring the urine protein creatinine ratio is useful to define the magnitude and, for this reason, the impact of proteinuria. Cardiac disease and other extrarenal issues can possibly trigger a transitory mild pre-renal proteinuria through increased glomerular permeability (7, 44).

No specific markers for CvRD are presently available, and their research, together with that of markers for CRS and for novel renal damage biomarkers, is a topic which can be substantial and of significant interest in both man and animals (14).

### Blood Culture

Endocarditis is a condition usually associated with bacteremia normally involving aortic and/or mitral valves; bacteria present in the blood flow directly contaminate the endocardial surface. Bacterial endocarditis can be reasonably diagnosed following more than two positive blood cultures (45–47).

The most commonly reported etiologic agents are streptococci, staphylococci, gram-negative bacilli, and *Bartonella* spp., while anemia, leukocytosis, thrombocytopenia, hypoalbuminemia, increased liver enzyme activity, and azotemia are frequent laboratory findings (48). Even with optimal sample collection, blood cultures may be negative in patients who are very strongly suspected of being bacteremic or having bacterial endocarditis, as shown by a retrospective study in which the causative organism was identified in only 58% of patients with endocarditis. One reason, in some cases, is reported as the possible prior administration of antibiotics before sample collection (8, 37, 48).

### Effusion Analysis

The abnormal collection of fluids within body cavities (effusion) is a possible finding during HF, and ascites (fluids in the abdomen) is seen more frequently with right-heart failure in dogs, as a consequence of acquired diseases (e.g., tricuspid regurgitation due to endocardiosis, heartworm disease, dilated cardiomyopathy, pericardial effusions, restrictive pericarditis) and congenital heart diseases (e.g., tricuspid dysplasia, ventricular and atrial septal defect) (49, 50). The analysis of these liquids can help in framing the ongoing pathological process.

The classifications of effusions, as recently proposed accordingly to underlie etiology, are clinically valuable and help the clinician to address to more specific diagnostic tests. Depending on the estimated total protein concentration and cytological criteria, effusions can be divided into four different groups: (a) transudate: can be rich or poor in protein; (b) exudate: can be septic or non-septic; (c) effusion caused by damaged vessels or viscus; and (d) effusion caused by cell exfoliation. Neoplastic outflow and reactive mesothelial hyperplasia are included in this last subgroup (51–53).

Protein-rich transudates due to increased hepatic or pulmonary intravascular hydraulic pressure may be caused by CHF and portal hypertension. Hallmarks of protein-rich transudates are a cloudy aspect, hemorrhagic aspect (but can also be clear), total protein (TP) concentration ≥ 2 g/dL, and presence of a variable number of neutrophils, lymphocytes, macrophages, and mesothelial cells. Effusions from cell exfoliation could derive from cardiac neoplasia as lymphoma, mesothelioma, carcinoma, and sarcoma (TP ≥ 2 g/dL). The term neoplastic effusion should be reserved for those fluids in which a neoplastic cell population has been evidenced (51–54).

### Vector-Borne Diseases

Canine leishmaniasis is induced by the protozoan parasite *Leishmania infantum*. It is a systemic pathology with changeable clinical symptoms that can be diagnosed with IFAT, ELISA, real-time PCR, or direct visualization of the parasite through a microscope (55, 56). Cardiac involvement, associated with myocardial damage, has been reported postmortem, with histopathology, in some affected dogs (57, 58), and also by evidencing elevated values of cardiac troponin I (cTnI) in diseased patients (59).

The cause–effect connection in such dogs was initially hypothesized as a direct consequence of a primary myocardial damage due to azotemia; however, this thesis was disavowed, as high cTnI levels were diagnosed in leishmaniotic patients with normal renal azotemia while lower cTnI levels were found in cases with idiopathic CKD (60).

A research has evaluated the cardiotoxic effects of meglumine antimoniate for 60 days at therapeutic dosage in dogs with leishmaniasis, and it revealed that there was no demonstration in cTnI dosage or electrocardiographic characteristics of cardiac damage (61).

Ehrlichiosis is a canine vector-borne illness (tick) primarily induced by *Ehrlichia canis*, a gram-negative intracellular bacteria. IFAT, ELISA, and real-time PCR are diagnostic tests most frequently used to diagnose ehrlichiosis.

The mix of vasculitis, myocardial hemorrhages, and hypoperfusion accompanied by a strong inflammatory reaction participates in the pathophysiology of heart damage in dogs with ehrlichiosis. Furthermore, *E. canis*-infected dogs showed higher serum cTnI values than controls did (62, 63). In contrast, myocardial damage in canine monocytic ehrlichiosis and modification in serum cTnI or clinically important alterations in ECG or in echocardiographic parameters were not recorded in experimentally induced acute and subclinical disease (64).

## Innovative Laboratory Test

### Cardiac Biomarker

A biomarker would supply information concerning analysis, prognosis, or response to treatment that is otherwise not readily obtainable using usual testing; consequently, a cardiac biomarker can be considered as a substance that is heart specific and that is released during a disease status, proportionally to the extent of damage (65). The two most investigated cardiac biomarkers in dogs are cTnI and 2 different forms of B-type natriuretic peptide (BNP).

#### Cardiac Troponin

Cardiac troponin I (cTnI), along with troponin-T (cTnT) and troponin-C, forms an aggregation of 3 myocardial proteins that are associated with the actin backbone inside myocardiocytes. Cardiac troponin I is a protein characteristic of cardiomyocyte involved in heart contraction and relaxation. It is attached to the actin filament, and it is present in moderate amount in the cytoplasm; therefore, the damage to cardiomyocytes leads to an extracellular release of cTnI (66–68); for this reason, an increase in its concentration may suggest a cardiac damage. Nevertheless, interestingly, Greyhounds and Boxers may possibly have inherently higher cTnI concentrations than other breeds (67–70).

In human patients, little to no cardiac troponin is noticeable in blood. Quantification of cardiac troponins may facilitate in quantifying the level of myocardial injury, supervise progression of diseases, and provide prognostic information in heart seizure patients. After a cardiac insult, an increase in these molecules might be found after 2–3 h, and a peak concentration is commonly reached between 18 and 24 h (71–75). Severe myocardial infarction in canine is not common as it is probably due to the rare occurrence of atherosclerosis in dogs (67, 76, 77). Nevertheless, chronic heart diseases like canine MVD and DCM are relatively common. Unfortunately, different causes might limit the utilization of cardiac troponin for veterinary patients; indeed, it is a sensitive but not specific marker. Cardiac troponin is partly removed by renal excretion; therefore, kidney disease can result in false increments (67, 78, 79).

In dogs, cardiac troponins might be released into the bloodstream in reaction to inflammation. High cTnI concentrations have been detected in dogs with MVD, and this increase was proportional to the severity degree of the MVD. Many substantial associations of age, CRP, heart rate, and left ventricular end-diastolic diameter on cTnI concentration were also present (67, 80).

MVD is not the only disease that has been associated with myocardial injury and increased troponins; among these, there are subaortic stenosis, pulmonic stenosis, DCM, arrhythmogenic right ventricular cardiomyopathy, myocarditis, dirofilariasis, cardiac hemangiosarcoma, and pericardial effusion (69, 80–93).

Myocarditis is a condition that recognizes different etiologies, such as *Leishmania infantum, Ehrlichia canis, Borrelia burgdorferi, Staphylococcus aureus*, and does not recognize a specific course (59, 94). A study has found 6 dogs with myocarditis seropositive for *Borrelia burgdorferi* and increased amounts of cTnI (94).

Myocardial injury associated with increased cTnI has also been described in many non-cardiac diseases such as pancreatitis, pyometra, parvoviral enteritis, leptospirosis, leishmaniasis, babesiosis, and ehrlichiosis (67).

#### Natriuretic Peptides

Natriuretic peptides are hormones generated inside and released from the ventricular and atrial myocardium. Mechanical stress and cardiac muscle stretching are causes of prohormones released from the cardiac muscle (79). Stress or stretch of the myocardium, in response to volume excess or other conditions, increases the generation of prohormones pro B-type natriuretic peptide (proBNP) and pro-atrial natriuretic peptide (proANP). The prohormones can be divided into inactive N-terminal fragments (NTproANP or NTproBNP) and active C-terminal fragments (ANP and BNP) (79).

Active atrial natriuretic peptide (ANP) and BNP are involved in cardiovascular homeostasis as they inhibit the renin–angiotensin–aldosterone mechanism, encourage vasodilatation by promoting natriuresis and diuresis, and reduce arterial blood pressure (1). BNP and NT-proBNP are increased in a very wide range of heart diseases, including MVD, DCM, and HCM (79, 81, 95, 96). Increased concentrations are also present in non-cardiac diseases but can affect the heart, such as in PH (97). More precisely, a high concentration of NT-proBNP can be suggestive of HF, whereas a low concentration is most consistent with a non-cardiac disease (78). NT-proBNP assay can be used for detecting dogs with occult DCM if combined with Holter monitoring with a sensitivity of 94.5% and specificity of 87.8% (98).

Very interestingly, there are also several studies revealing a correlation between NT-proBNP and survival time in dogs with MVD (99, 100).

The reference intervals of cardiac biomarkers (as for all laboratory tests) may vary depending on the type of test used, sensitivity, and working conditions; therefore, it is hard to define a unique value of these. Some reviews have provided reference intervals for the various tests available (67, 68, 79, 101).

### Proteomics

Proteomics is the study of the “proteome,” that is, the whole of the protein, as for example in a specific district of the body or tissue (102). Cardioproteomics in humans has recently been introduced as a possible way to investigate cardiovascular diseases (103).

Around proteomics, there is growing interest as it has proven to be useful for clearing complex cellular and molecular phenotypes and has shown to have some possibility to detect circulating biomarkers and drug targets for therapeutic intervention. Although cardioproteomics could still be considered as a recent application of proteomics, interesting and promising results have already been achieved, in both humans and dogs. A recent study aiming at finding new biomarkers, by a proteomic approach, for mitral regurgitation due to mitral valve prolapse in humans showed that haptoglobin, platelet basic protein, and C4b levels could be reduced in these subjects, and therefore, a possible role in the pathophysiology of mitral valve prolapse/mitral regurgitation has been hypothesized for these molecules (and pathways in which are involved) (104).

As already said, proteomics also stimulates the interest of veterinary research and different substrates and diseases are increasingly being investigated (105, 106). In Cavalier King Charles Spaniels, a study on serum proteins evidenced different expressions in healthy dogs when compared with those in patients suffering from MVD, from mild to severe degrees. Complement factor H isoform 2, calpain-3 isoform X2, dystrobrevin beta isoform X7, and CD5 molecule-like and hydroxyglutarate dehydrogenase showed to be reduced in mildly affected patients when compared to controls. All these proteins, with the exclusion of complement factor H, decreased accordingly to disease progression, showing in this way their potential in diagnosing and monitoring the disease (107).

### Microbiota

Intestinal microbiota (the set of *bacteria, fungi, archaea, viruses*, and *protozoa*) plays an important role in both health and disease states, regarding not only the gastrointestinal environment but also other districts of the body. Animal models play an important role in understanding the significance of gut microbiome configuration in immune system growth and its own relationship with health and disease (108). Furthermore, altered structure of gut microbiota, also called dysbiosis, participates in the genesis of some illnesses of the host (109).

In patients with HF, intestinal function and role are abnormal due to microcirculatory disorders, and it could be evidenced in a rise in pathogenic bacteria in feces, and in the number of mucosal adherent bacteria in the colon, which is also linked to an increase in intestinal permeability (110, 111). Patients with HF recognize significantly altered gut microbiota, and its composition in older HF patients is dissimilar from that of younger HF patients (112). Similarly, hypertension, one of the most relevant predisposing factors to cardiovascular disease, has been linked to microbiota (113).

Unfortunately, this aspect is almost ignored in canine medicine, but as shown by a recent pilot study on a selected fecal bacterial group in dogs suffering from cardiovascular diseases, suggesting possible differences between diseased and healthy dogs, it represents a very promising field of study (114).

## Conclusion

This manuscript reviews the published data regarding the importance of laboratory tests and biomarkers in cardiac diseases. The aim of this manuscript is to underline that traditional laboratory tests and biomarkers are significantly efficient tools for the management of patients with cardiac diseases. In addition, new frontier studies concerning proteomics and microbiota in heart diseases have also been reviewed.

## Author Contributions

AG, MC, GR, and VT contributed conception and design of the study. AF revised the section relative to cardiac disease. AG wrote the first draft of the manuscript. AS, BT, and AM provided critical revision. All authors contributed to the article and approved the submitted version.

## Conflict of Interest

VT was employed by company IDEXX. The remaining authors declare that the research was conducted in the absence of any commercial or financial relationships that could be construed as a potential conflict of interest.

## Abbreviations

ANP: Atrial natriuretic peptide
AST: Aspartate aminotransferase
BNP: B-type natriuretic peptide
CRP: C reactive protein
cTnT: Cardiac troponin-T
CRS: Cardio renal syndrome
CvRD: Cardiovascular–renal disorders
CHF: Chronic heart failure
CKD: Chronic kidney disease
CVD: Chronic valvular disease
CAD: Coronary artery disease
CK: Creatine kinase
DCM: Dilated cardiomyopathy
GFR: Glomerular filtration rate
HF: Heart failure
Hb: Hemoglobin
NTproANP or NTproBNP: Inactive N-terminal fragments
LDH: Lactate dehydrogenase
MVD: Mitral valve disease
PCV: Packed cell volume
NT-proBNP: Pro B-type natriuretic peptide
PH: Pulmonary hypertension
RDW: Red blood cell distribution width
RI: Renal insufficiency
SDMA: Symmetric dimethylarginine
USG: Urine specific gravity.
